# Pattern separation involves regions beyond the hippocampus in non-demented elderly individuals: A 7T object lure task fMRI study

**DOI:** 10.1162/imag_a_00404

**Published:** 2024-12-19

**Authors:** Zhengshi Yang, Xiaowei Zhuang, Katherine A. Koenig, James B. Leverenz, Tim Curran, Mark J. Lowe, Dietmar Cordes

**Affiliations:** Cleveland Clinic Lou Ruvo Center for Brain Health, Las Vegas, NV, United States; Imaging Institute, Lerner Research Institute, Cleveland Clinic, Cleveland, OH, United States; Cleveland Clinic Lerner College of Medicine, Cleveland, OH, United States; Department of Psychology and Neuroscience, University of Colorado, Boulder, CO, United States

**Keywords:** pattern separation, hippocampus, mnemonic similarity task, frontoparietal network, functional lateralization

## Abstract

Investigating the mechanism of differentiating similar representations, known as pattern separation, has primarily focused on the hippocampus. The roles of cortical regions and their interaction with the hippocampus remain largely unclear. In this study, we address this topic by analyzing whole-brain, high-resolution mnemonic similarity task (MST) fMRI data collected with a 7-Tesla MR scanner. Structural and functional MRI data were acquired from 55 non-demented elderly subjects. During the encoding phase of the MST task, participants responded with “indoor” or “outdoor” to 66 everyday objects. In the recognition phase, participants were asked to make “same” / “similar” / “new” judgments about objects that were either the same as previously seen objects (targets), similar but different from previously seen objects (lures), or new objects (foils). A general linear model was conducted on hippocampal regions of interest (ROIs) and at the whole-brain level with five conditions, including “new” response to foils (foil), “same” response to targets (hit), correct “similar” response to lure (lure correct rejection, LureCR), false “same” response to lure (lure false alarm, LureFA), and one condition for all others. The activity difference when lures were identified as “similar” compared to “same” (LureCR vs. LureFA) was used to assess if a region is involved in pattern separation. An association analysis was conducted to test if lure discrimination performance was correlated with activity difference of hippocampal ROIs between LureCR and LureFA, as well as age. Task-based functional connectivity between hippocampal ROIs and other regions involved in pattern separation was examined. In the hippocampal ROI analysis, left anterior CA3/DG showed greater activity in LureCR condition compared to LureFA. All other hippocampal ROIs did not show differential activity. Better lure discrimination performance was associated with larger activity difference between LureCR and LureFA at left anterior CA3/DG and right anterior subiculum. In the whole-brain analyses, regions in the frontoparietal network (FPN) consistently showed increased activity in the Hit, LureCR, and LureFA conditions, and the activity was right-lateralized for Hit and LureFA conditions but bilateral for LureCR condition. Eleven clusters, mainly located in the left hemisphere, were identified to show significant activity difference between LureCR and LureFA condition, including left FPN, middle temporal lobe, and subcortical regions. In summary, with the whole-brain high-resolution MST fMRI data, regions exhibiting the pattern separation signature were found to be lateralized to the left hemisphere in elderly participants. The left and right FPN are suggested to have distinct functional roles in the MST. The right FPN contributes to retrieving previously viewed same or similar objects, while the left FPN is preferentially involved in pattern separation. Furthermore, the pattern separation process might require the coordinated effort of FPN and hippocampus, with their interaction potentially mediated by subcortical regions.

## 1 Introduction

Pattern separation is the cognitive mechanism through which unique memory representations are created to differentiate between similar, yet distinct stimuli. This process plays a crucial role in episodic memory, enabling the brain to encode and retrieve specific events and experiences with precision. (Sahay et al., 2011). As the complementary process of pattern separation, pattern completion describes the process of retrieving previously stored memory representations when incomplete or degraded representations are given (Marr et al., 1991;Rolls, 2013). The importance of the hippocampus on pattern separation and completion has been emphasized in computational models since 1991 (Marr et al., 1991;Norman & O’Reilly, 2003). Animal studies revealed compelling evidence of distinct and specialized functions of hippocampal subfields in the pattern separation and completion process with both rodents (Sakon & Suzuki, 2019) and monkeys (Leutgeb et al., 2007;Neunuebel & Knierim, 2014). The circuit from entorhinal cortex to dentate gyrus (DG) to CA3 is frequently implicated in pattern separation (Leal & Yassa, 2018;Yassa & Stark, 2011).

Consistent with animal studies (Yassa & Stark, 2011), human functional MRI (fMRI) research revealed distinct roles of hippocampal subfields in pattern separation and pattern completion (Bakker et al., 2008;Berron et al., 2016). In multiple studies, high-resolution fMRI data were collected to examine their distinct activities (Bakker et al., 2012;Kirwan & Stark, 2007;Lacy et al., 2011;Stevenson et al., 2020) when the participants were performing a mnemonic similarity task (MST). In this task, participants were asked to judge whether the presented stimuli were the same as previously seen objects (targets), similar but different from previously seen objects (lures), or new items (foils) (Stark et al., 2019). The differential activity between correct “similar” response to lure stimuli (“similar” | lure; LureCR) and false “same” response to lure stimuli (“same” | lure; LureFA) indicates a region’s involvement in pattern separation (Stark et al., 2013;Stevenson et al., 2020). Using high-resolution fMRI data, CA3/dentate gyrus (CA3/DG) was the region most frequently reported to be engaged in pattern separation, which was in line with animal studies (Leutgeb et al., 2007;Neunuebel & Knierim, 2014). The high spatial resolution came with the cost of limited spatial coverage focusing on medial temporal lobe in most cases. Even with high-resolution fMRI data, CA3 cannot be confidently isolated from DG, thus CA3 and DG are usually combined for the analysis.

Besides the hippocampus, other brain regions might also be involved in pattern separation to create distinct memory representations. To investigate the role of regions outside the hippocampus, whole-brain MST fMRI data were collected with a compromised spatial resolution in previous studies (Bowman & Dennis, 2016;Motley & Kirwan, 2012;Pidgeon & Morcom, 2016;Wais et al., 2017). In the condition that lure trials were correctly rejected, lateral prefrontal cortex and bilateral parietal regions showed increased activity, together with increased functional connectivity between frontoparietal network and hippocampus (Bowman & Dennis, 2016;Pidgeon & Morcom, 2016;Wais et al., 2017). From these studies, regions outside the hippocampus, including inferior frontal gyrus and lateral parietal lobe, were detected to show activity consistent with the pattern separation process. It is speculated that pattern separation is fundamental to many aspects of cognition, instead of merely memory, and other cortical regions might also be involved in pattern separation (Kent et al., 2016). These whole-brain fMRI studies suggested that the cognitive control process, as a central role of frontoparietal network, might affect the decision-making in the MST by coordinating with the cognitive processes occurring in the hippocampus (Moscovitch, 1992). But the low spatial resolution (about 3 mm x 3 mm x 3 mm) made it challenging to delineate the activity of hippocampal subregions, thus it is infeasible to capture the pattern separation signature of hippocampal subregions and regions beyond the hippocampus at the same time (Yassa & Stark, 2011). In addition, a conceptual model of mnemonic interference hypothesizes that the representational overlap between two similar inputs incrementally decreases as information projecting from domain-specific visual streams into the hippocampus, leading to orthogonalized activity in CA3/DG (Reagh & Yassa, 2014). To the best of our knowledge, whether the regions at the downstream of hippocampal CA3/DG preserve the orthogonalization and which regions are involved largely remain unclear.

A few recent studies used accelerated acquisition technique (Xu et al., 2013) on 3T MR scanners to collect high-resolution whole-brain fMRI data among healthy young adults (Klippenstein et al., 2020;Nash et al., 2021). In a study where participants made two-button responses (“same” or “new”) to target, lure, and foil stimuli, differential activity of “same” | target condition compared to “new” | lure condition was observed in the hippocampus and occipital lobe, although the activity difference when participants made correct vs. false response to lure stimuli (i.e., “new” | lure vs. “same” | lure) was not tested (Klippenstein et al., 2020). The medial temporal lobe is sensitive to susceptibility artifacts, leading to signal loss and geometric distortion in fMRI (Reznik et al., 2023). Although within-slice acceleration could alleviate the distortion, accelerated fMRI acquisition technique had a lower temporal signal-to-noise ratio (tSNR) compared to standard fMRI acquisition protocols (Risk et al., 2021;Srirangarajan et al., 2021). These factors, together with long TR (TR = 3s and stimulus duration = 3s), could potentially undermine the capability of high-resolution fMRI data to detect neural activity related to pattern separation (Nash et al., 2021). More studies are still required to identify regions beyond hippocampus engaged in pattern separation and their interaction with hippocampal subregions.

The ability to differentiate similar representations (items or events) is one important aspect of episodic memory. Older age and neurodegenerative diseases were associated with deteriorated episodic memory (Tromp et al., 2015). The elderly subjects are more likely to respond “same” to the lure stimuli compared to the younger population but have no difference in judging repeated stimuli as “same”, which suggests a deficit in pattern separation together with a concurrent bias toward pattern completion (Berron et al., 2018;Stark et al., 2015;Vieweg et al., 2015;Yassa & Stark, 2011). Among a group of older adults but not young adults, reduced activation in the medial temporal lobe was associated with worse object mnemonic discrimination (Berron et al., 2018). A separate study showed that hyperactivity in the CA3/DG was associated with behavioral deficit across both older and young adults, although such an association was not replicated within older adults or young adults (Yassa & Stark, 2011). Collectively, the association between behavioral deficit in pattern separation and functional activity in the hippocampus remains unclear. Investigating the activation of hippocampal subfields and its association with behavioral performance in MST among an elderly cohort with a larger sample size would be beneficial for validating previous findings. Until now, there has been a lack of whole-brain high-resolution fMRI data to evaluate brain activity involved in pattern separation among elderly populations.

In this study, we collected whole-brain fMRI data from a group of elderly adults on a Siemens 7T MR scanner using an accelerated acquisition technique to achieve both high spatial resolution (1.5 mm isotropic) and reasonable temporal resolution (1.53 seconds). Although the within-slice acceleration used in the study could lead to lower tSNR compared to the standard fMRI acquisition technique, the higher magnetic strength could compensate for the loss of sensitivity compared to lower field studies (Risk et al., 2021;Srirangarajan et al., 2021). The fMRI data collected from a 7T scanner could have up to 300% improvement of tSNR in cortical area compared to a 3T scanner (Morris et al., 2019). Although the actual tSNR gain in the hippocampus has not been comprehensively examined as in cortical areas, the 7T data is still expected to provide a more robust activation pattern than the 3T data.

In this study, we investigated the differential activity between LureCR and LureFA condition in the hippocampal ROIs and at whole-brain level. The association of participants’ lure discrimination performance with hippocampal activation difference between LureCR and LureFA, as well as age, was evaluated. We hypothesized that CA3/DG would have discriminative activity between LureCR and LureFA. Based on previous whole-brain fMRI studies, the regions located in the frontoparietal network (FPN) were hypothesized to show pattern separation signature and have significant functional connectivity with hippocampal ROIs.

## 2 Methods

### 2.1 Subjects

Sixty-two non-demented elderly subjects were initially recruited for the study from the Cleveland Clinic at Cleveland, Ohio since 2022. Before collecting MRI data, all participants underwent an outside-scanner training session and a mock run of our task. Only participants with a no-response rate less than 20%, and correct response rates greater than 60% for target and foil stimuli proceeded to MRI scan. Among the initially recruited participants, four were excluded due to inability to complete the task training session, one was excluded due to severe claustrophobia in the 7T environment, and two were excluded due to extremely enlarged ventricles on the structural MRI leading to failed co-registration between functional and structural scans. A final cohort of 55 participants (age range 61–81 years; 23 male and 32 female; 3 African American, 45 non-Hispanic White and 7 Hispanic White) were included in our study, consisting of 40 cognitively normal participants and 15 patients with mild cognitive impairment (MCI). This study was approved by Cleveland Clinic Institutional Review Board. All participants have given written, informed consent for their participation. The subjects’ consent was obtained according to the Declaration of Helsinki.

### 2.2 MRI acquisition

**Fig. 1.**
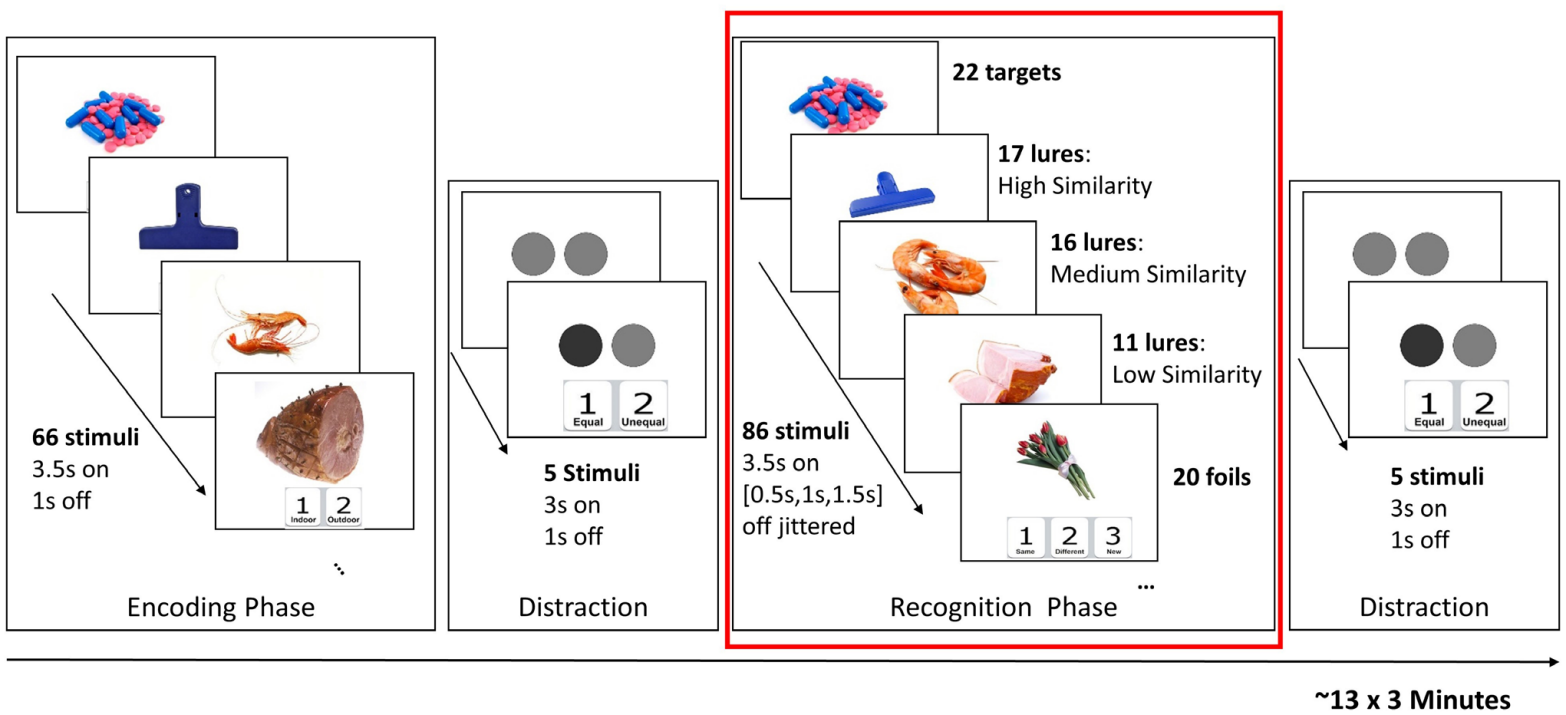
Task paradigm of the MST. Each MST fMRI run lasts approximately 13 minutes; it consists of one encoding phase, one recognition phase, and two distraction phases. An instruction screen was presented for 5 seconds prior to each phase (encoding, distraction, recognition) to remind participants of the upcoming task. Each participant underwent three consecutive MST fMRI runs within a single fMRI session. This study is focused on the recognition phase (marked in red) to investigate brain regions involved in the pattern separation process.

The imaging data were acquired on a 7T Siemens MAGNETOM Terra MR scanner, using a 32-receive channel/8 transmit channel head coil at the Mellen Center of the Cleveland Clinic, Cleveland, Ohio. Standard B1 shimming without parallel RF transmission was used. Patient specific shimming did not provide significant advantage in image quality for the EPI sequence used in the study. Advanced B0 shimming was used as provided with the Terra user interface. The whole-brain T1-weighted 3D MP2-RAGE structural scans were acquired with 0.8 mm isotropic voxel size, field of view = 227 mm x 240 mm x 186 mm, 288 slices, TR = 6000 ms, TE = 2.89 ms, TI 1 = 800 ms, flip angle 1 = 4 degrees, TI 2 = 2700 ms, flip angle 2 = 5 degrees, with the acquisition time of 5 minutes 58 seconds. The high-resolution hippocampus 3D T2-weighted structural scans were acquired with the orientation perpendicular to the long axis of the hippocampus, TR = 8040 ms, TE = 76 ms, flip angle = 60 degrees, 55 slices, in-plane resolution 0.44 mm x 0.44 mm, slice thickness 1 mm with a 0.1 mm gap, and FOV = 224 mm x 60 mm x 224 mm, with the acquisition time of 7 minutes 48 seconds. Each functional MRI run was collected using a multi-band gradient echo EPI sequence: TR = 1530 ms, TE = 22 ms, flip angle = 70 degrees, phase-encoding direction = anterior to posterior, 1.5 mm isotropic voxel size, 81 slices, FOV = 192 mm x 192 mm x 122 mm, GRAPPA acceleration (iPAT) = 2 (24 reference lines), slice acceleration = 3, phase partial Fourier 6/8, BW 1776 Hz/px, echo spacing 0.67 ms, and 510 time points, with a duration of 13 minutes 13 seconds. The spatial resolution of 1.5 mm was previously demonstrated to be able to detect distinct activity between hippocampal ROIs (Stevenson et al., 2020;Yassa, Lacy, et al., 2011). Considering the event-related design of the task and the short duration of each trial, we did not sacrifice the temporal resolution to achieve higher spatial resolution. Three fMRI runs were acquired from each participant. In addition, standard GRE field mapping sequences were collected for correcting the field inhomogeneity. The mean fMRI volumes and the gray plots of time series from two subjects were shown inSupp. Figure 1.

### 2.3 Object lure task

The object lure task used in the study was a modified version (detailed later) of the*Mnemonic Similarity Task*(MST) (Stark et al., 2013;Yassa, Mattfeld, et al., 2011). This task consisted of an encoding phase, a memory recognition phase, and two distraction phases immediately after encoding and recognition phases (Fig. 1). An instruction screen was presented for 5 seconds prior to each phase (encoding, distraction, recognition) to remind subjects of the upcoming task.

#### 2.3.1 Encoding phase

Sixty-six 400 x 400-pixel objects were shown sequentially at the center of the screen during the encoding phase. Participants were engaged in an explicit encoding task. Each object (i.e., stimulus) was presented for 3 seconds on a white background. The inter-stimulus interval was 1 second (white background only). To encourage successful encoding of object details, the encoding task instructed the participant to press a button to indicate whether the object was an indoor object (button 1 = indoor) or an outdoor object (button 2 = outdoor) after each item was presented. Although the indoor/outdoor task could be considered incidental to the goal of remembering the stimuli, we referred to it as “explicit” because, during the initial outside-scanner training session and a mock run of our task, it was made clear to participants that objects from the indoor/outdoor task would appear on a later recognition task.

#### 2.3.2 Distraction phase

Participants were asked to judge whether two circles on the screen were equally dark in shade (button 1 = equal, button 2 = unequal). During each distraction phase, five stimuli were presented, with the same 3 seconds duration and a 1 second inter-stimulus interval. A distraction phase between encoding and recognition is designed to clear participants’ mind from the encoding task. Both distraction phases additionally provided baseline activation for the brain without performing the memory task.

#### 2.3.3 Recognition phase

During the recognition phase, 86 objects were presented sequentially at the center of the screen, including 22 targets, 44 lures, and 20 foils. Participants were instructed to decide if the current stimulus was the same (button 1 = “same”) as in the encoding phase, similar but different (button 2 = “similar”) from the one in the encoding phase, or a new stimulus not seen before (button 3 = “new”). The objects used in this task and the similarity levels of lure objects were obtained from the original MST (Stark et al., 2013;Yassa, Mattfeld, et al., 2011) and are publicly available from Dr. Stark’s lab. The lures consist of objects of high (LureH, 17 stimuli), medium (LureM, 17 stimuli), and low (LureL, 11 stimuli) similarity levels, corresponding to lure 3, 4, and 5 in the original MST. Lure 1 and 2 in the original MST are more like previously viewed objects. Considering that elderly adults require larger changes to successfully encode new information as distinct from previously learned information (Yassa & Stark, 2011), lure 1 and 2 were not used to ensure that the elderly cohort in the study have sufficient correct responses for the analysis.

#### 2.3.4 Optimizing stimuli sequencing in the recognition phase

During the recognition phase, a total of 86 stimuli were presented and each stimulus was on for 3.5 seconds. We utilized a genetic algorithm to obtain the above-mentioned number of stimuli in each category (targets, lureL, LureM, LureH and foils), their optimum presenting orders, and the inter-stimulus interval (jittered among 0.5 second, 1 second, and 1.5 seconds) during the recognition phase. The objective here is to maximize the detection power (Cordes et al., 2012) over seven contrasts of potential interest: 1) “same” | target vs. distraction; 2) “similar” | lureL vs. distraction; 3) “similar” | lureM vs. distraction; 4) “similar” | lureH vs. distraction; 5) “new” | foil vs. distraction; 6) behavioral pattern separation contrast (“similar” | lure vs. “similar” | foil); and 7) recognition contrast: (“similar” | lure and “same” | target) vs. (“same” | lure and “similar” | foil). The notation “A|B” represents the response A (“same”, “similar”, or “new”) to a given stimulus type B (target, lure, or foil). In this optimization, we further assumed that 1) BOLD response could be modeled by the canonical hemodynamic response function; 2) perceived randomness was given; and 3) approximate probabilities for the possible conditions were available from the behavior data inStark et al. (2013). In general, a 22% increase in detection efficiencies was obtained after 500 generations in the genetic algorithm. We further computed the detection power based on the same design using the actual task accuracies from the 55 participants, and a 21% increase in power was observed.

Taken together, each participant underwent three consecutive MST fMRI runs within a single fMRI session. Each run lasts approximately 13 minutes, and different objects were used in these three runs. We developed a total of seven different object-related memory runs with different stimuli, with one used for training, three used during fMRI scanning, and three were backup runs. If one run failed inside the scanner, then the backup set was used for a repeated scan.

### 2.4 Delineating hippocampal ROIs

**Fig. 2.**
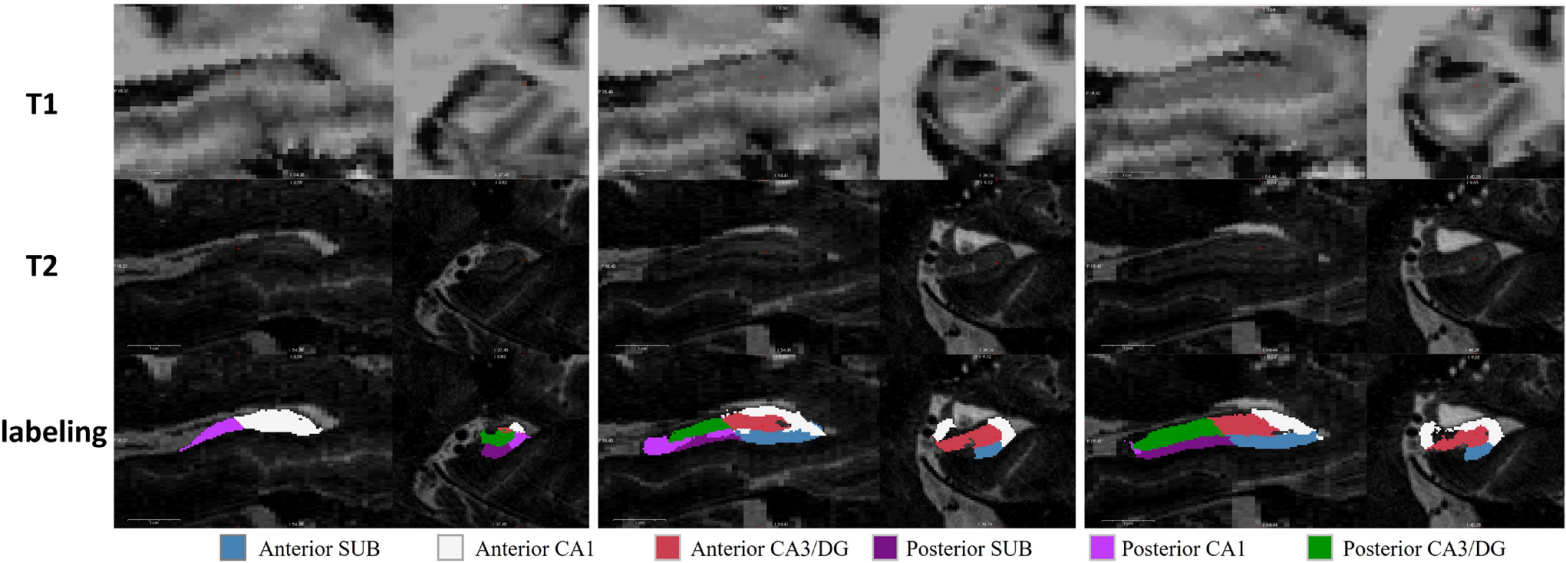
Sagittal and coronal view of ROIs in left hippocampus from a single individual. The labeling of hippocampal ROIs overlaid on the T2 image (bottom), together with T1 (top) and T2 images (middle), was shown in the figure.

The segmentation of hippocampal ROIs was carried out with the FreeSurfer software suite (v7.2,https://surfer.nmr.mgh.harvard.edu/). In detail, the T1 structural image was first analyzed with the main FreeSurfer command “recon-all” with default settings. With the output from the previous step, the command “segmentHA_T2.sh” was used to segment hippocampal regions with T2 image fed into the analysis. Recent MST fMRI studies showed that the anterior and posterior part of hippocampal subfields could behave differently during retrieval (Nash et al., 2021;Stevenson et al., 2020). Six hippocampal ROIs were defined, including anterior CA1, anterior subiculum, anterior CA3/DG, posterior CA1, posterior subiculum, and posterior CA3/DG. Left and right hippocampus were analyzed separately, leading to twelve hippocampal ROIs. The voxels within hippocampal head are defined as “anterior”, and the voxels within hippocampal body are defined as “posterior”. The hippocampal labeling from one example subject is shown inFigure 2. SPM12 (https://www.fil.ion.ucl.ac.uk/spm/software/spm12/) was applied on the T1 image to generate the gray matter (GM), white matter (WM), and cerebrospinal fluid (CSF) masks. Advanced Normalization Tools (ANTs,http://stnava.github.io/ANTs/) were used for co-registering fMRI data and T1 structural image. With the 7T functional MRI data collected in the study, it was observed that the participants’ T1 images were not well co-registered to their corresponding fMRI images by conducting a standard affine transformation between the mean fMRI and T1 image with the default settings. As shown inSupp. Figure 2, the ventricle was not well aligned between fMRI and T1 images, which might not be a major issue for a large ROI but could have a substantial effect on extracting hippocampal ROI time series, especially considering that the hippocampus is a small region and anatomically close to the ventricle. To address this issue, the affine transformation implemented in the study was carried out with a dilated GM+WM mask (Yang et al., 2023). The dilated GM+WM mask was generated by first combining the gray matter and white matter masks and then dilating the combined mask by two voxels. The GM and WM mask could avoid the potential detrimental effect of non-brain voxels in the co-registration process. The dilation process was required to include the boundary between ventricle and its neighboring voxels in the mask to better co-register the ventricle between these two imaging modalities in the revised affine transformation approach, which was beneficial because of the stronger contrast between ventricle and its neighboring voxels in the 7T MR scanner than in a lower-field MR scanner. Other neuroimaging toolboxes, for example FSL, have boundary-based registration process available, which might be an alternative approach to improve the co-registration, although it was not tested or compared in our study. Then, the labeling of hippocampal ROIs in T1 space from FreeSurfer was transformed to individual fMRI space by using the inverse affine transformation matrix.

### 2.5 Whole-brain voxel-wise analysis and hippocampal ROI analysis

The original functional MRI time frames were first slice-timing corrected in SPM12 (http://www.fil.ion.ucl.ac.uk/spm/). Before realignment, the 7T field inhomogeneity was estimated using the SMP12 field map toolbox (SPM-FMT). More specifically, a voxel displacement map (VDM) was estimated from the two GRE field mapping sequences in SPM-FMT, and the estimated VDM was used with Realign & Unwarp in SPM12 for doing a combined static and dynamic distortion correction. All three task fMRI runs were realigned and unwarped together.

By utilizing the hippocampal ROI labeling in each individual fMRI space, we extracted the voxel-wise time series within each ROI and then averaged to derive the mean time series, which is used for the following analysis. Computing the hippocampal ROI time series in the original fMRI space could avoid the spatial interpolation of fMRI data and reduce the partial-volume effect. No spatial smoothing is applied in the process of deriving hippocampal ROI time series.

For the whole-brain voxel-wise analysis, Gaussian smoothing with full-width half-maximum (FWHM) as 4 mm was applied on fMRI data after distortion correction. Spatial smoothing is applied to improve the SNR and alleviates the imperfectness of spatial normalization to MNI template space, which is beneficial for group analysis. The smoothed data were then co-registered to the individual T1 image space by using the transformation matrix derived from the revised affine transformation approach (detailed in the previous section), and then spatially normalized to standard MNI space by using the symmetric diffeomorphic transformation in ANTs. A brief flowchart of the preprocessing pipeline is shown inFigure 3.

**Fig. 3.**
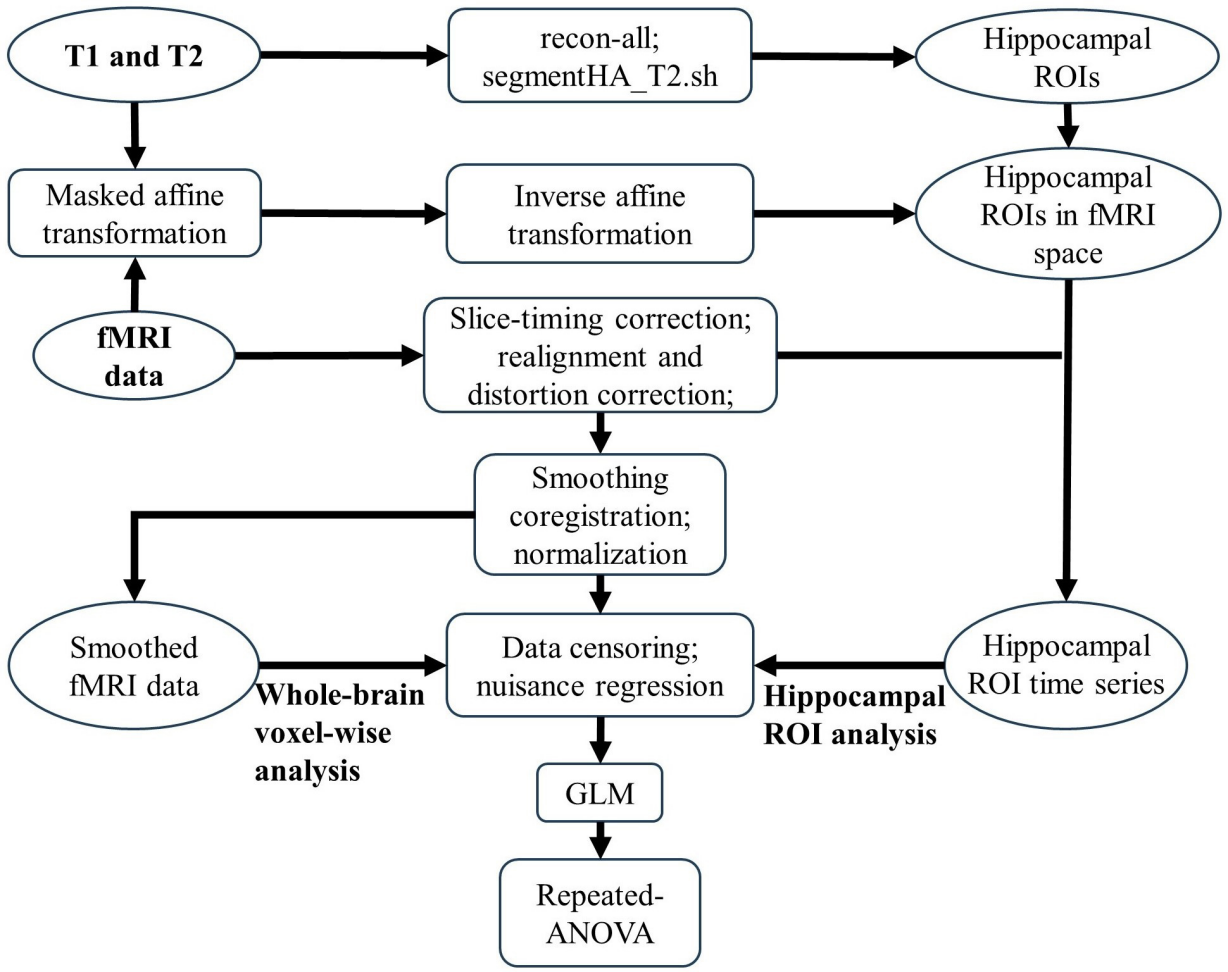
Flowchart of the processing steps for whole-brain voxel-wise activation analysis and hippocampal ROI activation analysis.

### 2.6 Statistical analysis

With the six-degree motion parameters derived from co-registration, framewise displacement for each time point was computed (Power et al., 2014). By averaging the framewise displacements of all time points for each participant, the mean framewise displacement ranges from 0.11 mm to 1.75 mm with median value as 0.29 mm. For both hippocampal ROI time series and whole-brain voxel-wise time series, the time points with framewise displacement greater than 1 mm and their immediately preceding and following time points were discarded as a data censoring procedure (Power et al., 2012). Only the functional MRI data collected during recognition phases were included in the analysis. A nuisance regression was then applied on the remaining time points of each fMRI run with the linear trend, six motion regressors and their first-order derivatives, three WM components, and three CSF components from aCompcor included as the regressors (Behzadi et al., 2007). The fMRI data after nuisance regression were converted to the percentage of signal change, concatenated across all three fMRI runs, demeaned, and standardized. The final output time series was then used in the following analysis.

The general linear model (GLM) was constructed with five conditions, including Hits (“same” | target), lure correct rejections (LureCRs, “similar” | lure), lure false alarms (LureFAs, “same” | lure), Foils (“new” | foil), and a collapsed regressor combing all other responses of no-interest and no-response trials. The same as previous studies, the Foil condition was treated as the baseline condition, and the estimated beta values were first subtracted from the beta value of the Foil condition before further statistical analysis (Nash et al., 2021;Stevenson et al., 2020). Note that splitting lures into high, medium, and low similarity groups led to fewer trials for each condition; some participants had very few or even no trials for a certain similarity group, which thereby led to unstable results. Because of this reason, the lure objects with different similarity levels were combined in the analysis. The same analysis was applied to the whole-brain and hippocampal ROI. Unless otherwise stated, the beta values reported in the*Results*are the values after subtracting the beta coefficient of the Foil condition. This step had no influence on the significance level of the activity difference between LureCR and LureFA in the following repeated analysis of variance (repeated-ANOVA), since the coefficients of both were subtracted by the same value.

The activity (i.e., beta value) for the left and right hippocampal ROIs was first estimated using the GLM model with the average time series from each ROI. Repeated-ANOVA was then used to test if a ROI had significantly different beta coefficients between LureCR and LureFA (without Hits condition). If a region shows a high pattern separation effect, its activity for lure and new stimuli is expected to be higher than the repeated items due to the repetition suppression effect (Kim et al., 2020). Instead of only considering the types of presented stimuli in the contrast, that is, activity for repeated < new = lure, recent studies (Nash et al., 2021;Stevenson et al., 2020;Yassa, Lacy, et al., 2011) adopted the contrast of LureCR vs. LureFA, which took participants’ responses into consideration. The applicability of this contrast on elderly population was previously demonstrated, which was particularly helpful for an elderly population because of their bias toward “same” responses (Yassa, Lacy, et al., 2011). Linear regression analysis was then used to evaluate how strongly the beta difference between the LureCR and LureFA conditions or age was associated with participants’ capability to correctly identify lures. Instead of directly using the percentage of lures with “similar” response, namelyP(“similar” | lure), as the metric to characterize participants’ performance in correctly identifying lures, the metricP(“similar” | lure)—P(“similar” | foil), namely the lure discrimination index (LDI) (Stark et al., 2019), was used in the analysis to correct for any bias the participant may have in using the “similar” response overall.

To identify the regions beyond the hippocampus involved in lure discrimination, GLM was applied on the spatially normalized fMRI data for voxel-wise analysis. Then, repeated-ANOVA was used to test the beta difference between LureCR and LureFA conditions (Hits condition is not included). Cluster-wise correction was used to identify significant clusters; a cluster with a minimum of 67 voxels at the significant level p < 0.001 was determined to be significant (α < 0.05) based on 3dttest++ cluster-wise correction algorithm in AFNI software suite (https://afni.nimh.nih.gov/). For each participant, the voxel-wise time series within these clusters (in the MNI space) were extracted and averaged to derive the mean time series of each cluster. The functional connectivity between hippocampal ROIs and these clusters was then characterized by Pearson’s correlation. One-sample t-test was applied after Fisher’s r-to-z transformation to examine if these clusters had significant functional connectivity with hippocampal ROIs during the task fMRI scan. Noted that this task-dependent functional connectivity is different from the intrinsic functional connectivity observed in resting-state fMRI data.

## 3 Results

### 3.1 Task performance

The proportions of “same”, “similar”, “new” response, and no-response for target, lure, and foil stimuli are shown inFigure 4. The participants correctly identified target as “same” and identified foil as “new” with accuracy above 70%. 52.5% of the lure stimuli were correctly identified as “similar”. Repeated-ANOVA showed that the correct response rates for target (P(“same” | target) = 79.3%/80.1%/78.4% for run 1/2/3) and lure (P(“similar” | lure) = 51.9%/50.5%/54.2% for run 1/2/3) objects did not show significant difference across three fMRI runs. The correct response rate for foil objects (P(“new” | foil) = 73.7%/68.4%/67.3% for run 1/2/3) is slightly lower in run 2 and 3 (p = 0.03).

**Fig. 4.**
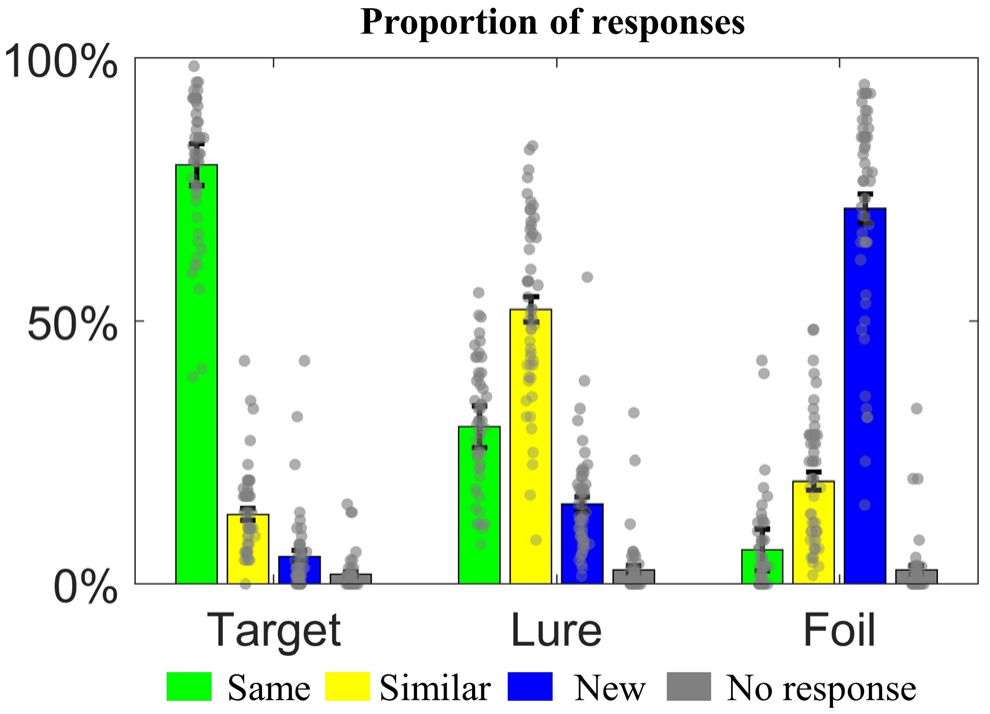
Participants’ performance in the MST task. The y-axis indicates the proportion of responses to target, lure, and foil stimuli. The error bar indicates the standard error. Each dot represents the proportion of responses over three fMRI runs from one individual.

### 3.2 Hippocampal ROI analysis

**Fig. 5.**
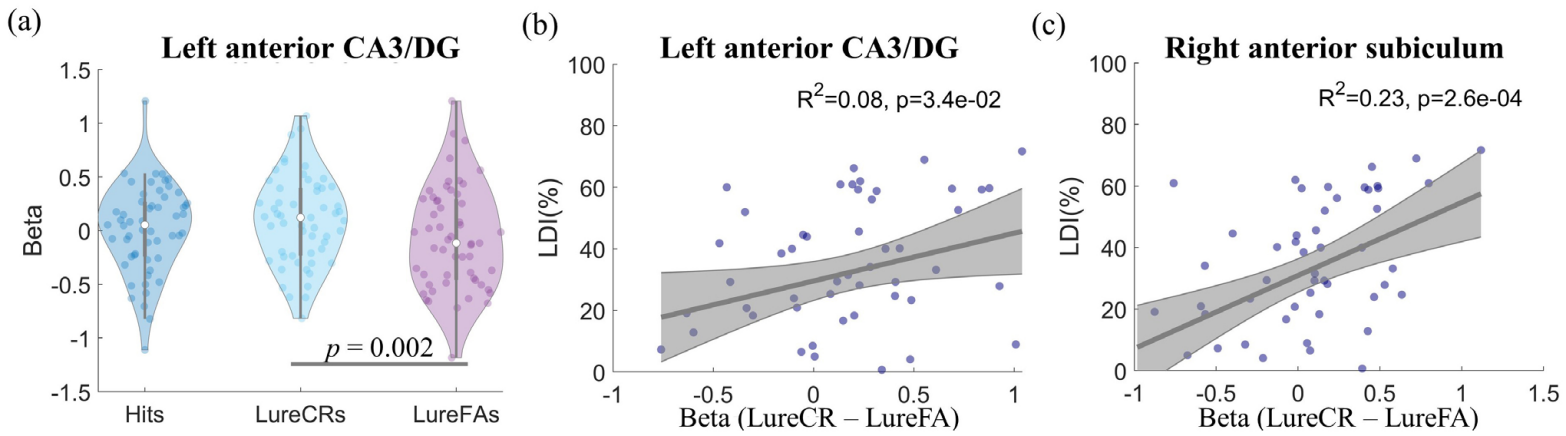
Hippocampal ROI analysis. (a) Violin plot of beta coefficients for left anterior CA3/DG. Only the left anterior CA3/DG had significantly different beta value between LureCR and LureFA. The violin plots for all other hippocampal ROIs can be found inSupp. Figure 3. The linear fittings between participants’ behavioral metric, namely the LDI, and beta difference between LureCR and LureFA at left anterior CA3/DG (b) and right anterior subiculum (c) are shown in the figure. Age and other hippocampal ROIs did not have significant association with the LDI.

The left anterior CA3/DG showed significantly different beta values between LureCR and LureFA condition after Bonferroni correction for multiple comparisons (α< 0.05), with greater activation for LureCR condition (Fig. 5a, F(1,52) = 10.1, uncorrected*p*= 0.002); all the other hippocampal ROIs did not show discriminative activity between LureCR and LureFA (Supp. Fig. 3). The beta differences between LureCR and LureFA at left anterior CA3/DG (R^2^= 0.08, p = 0.034,Fig. 5b) and left anterior subiculum (R^2^= 0.23, p = 0.00026,Fig. 5c) were positively associated with the LDI. Age and other hippocampal ROIs did not show association with the LDI (p > 0.05). The association between age and LDI was still not observed when only CN participants were included in the analysis.

### 3.3 Whole-brain analysis

The mean beta maps for Hit, LureCR, LureFA, and Foil conditions, before subtracting the beta value of the Foil condition, are shown inSupp. Figure 4. Strong activation in the occipital lobe was consistently observed in all four conditions, which was expected since all these conditions involve the reception of visual stimuli, suggesting that the task design correctly modeled the brain activation stimulated by these conditions. To better visualize brain activity closely related to memory retrieval, the Foil condition was treated as the baseline condition. The beta maps of the Hit, LureCR, and Lure FA conditions after subtracting the coefficient of Foil condition were shown inFigure 6. Regions within the frontoparietal network (FPN) consistently showed increased activity in the Hit, LureCR, and LureFA conditions. The activity was right lateralized for the Hit and LureFA conditions, but bilateral for the LureCR condition.

**Fig. 6.**
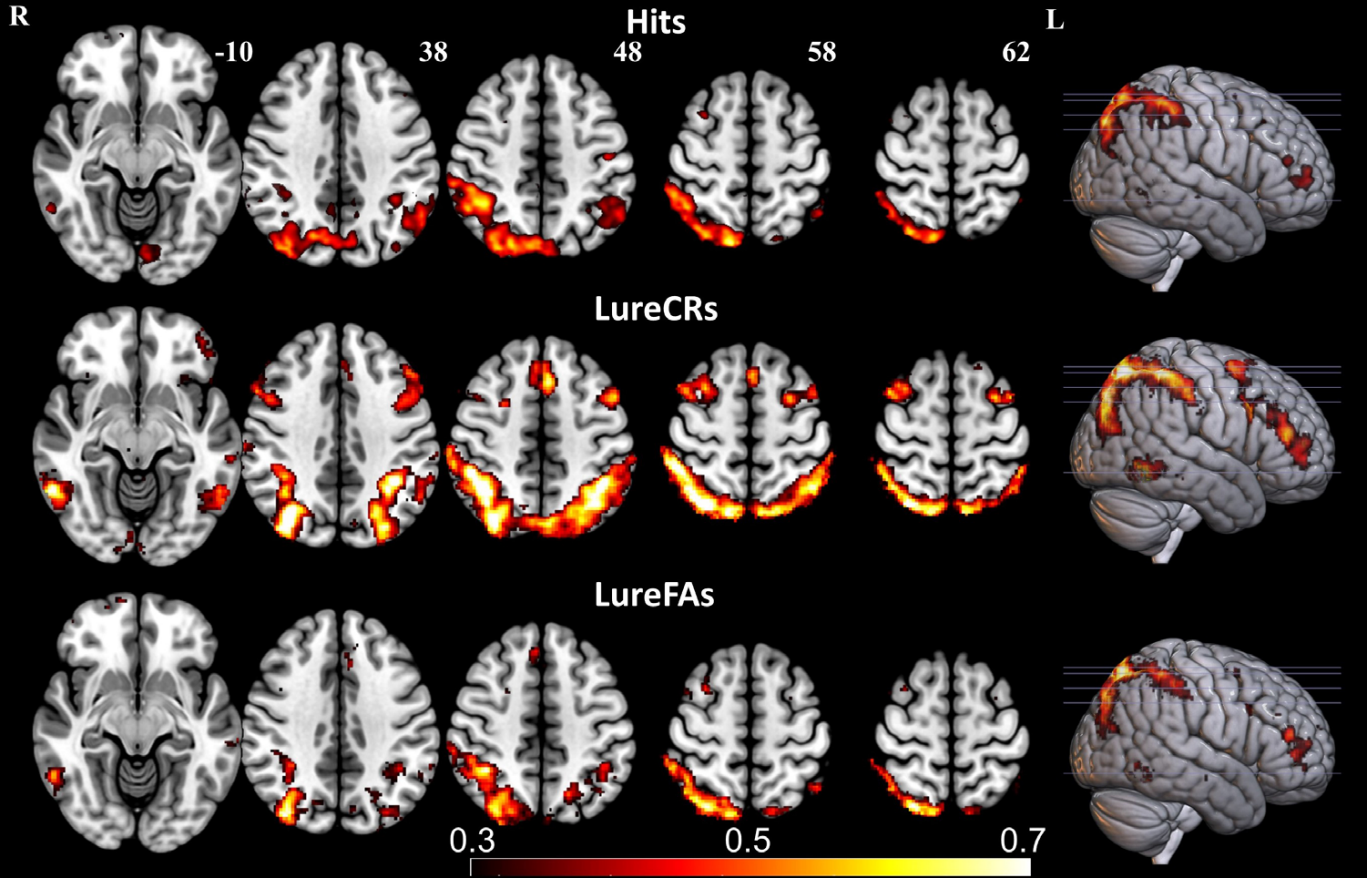
The beta maps for Hit, LureCR, and LureFA conditions. The Foil condition is used as the baseline condition. Hit and LureFA conditions have right-lateralized activity, and LureCR condition has bilateral activity. The activity is mainly located in frontoparietal regions.

Repeated-ANOVA was carried out to identify the regions showing discriminative activation between LureCR and LureFA condition. The difference between LureCR and LureFA was mainly located in the left hemisphere (seeFig. 7a). Eleven clusters were determined to be significant after cluster-wise correction, including left precentral gyrus (2983 voxels), left parietal lobe (2478 voxels), left supplementary motor area (861 voxels), right lingual gyrus (854 voxels), left middle temporal lobe (735 voxels), right postcentral gyrus (204 voxels), bilateral caudate nucleus (left: 168 voxels; right: 135 voxels), left precuneus (151 voxels), left thalamus (114 voxels), and right amygdala (84 voxels). The LureCR condition consistently had a higher beta value than the LureFA condition for all clusters. The violin plots of the beta coefficients for the two largest clusters, namely left precentral gyrus and left inferior parietal lobe, are shown inFigure 7band7c, and the plots for all the other clusters can be found in theSupp. Figure 5. One-sample t-test showed that all hippocampal ROIs had significant connectivity with at least one cluster. Bilateral posterior CA1 had relatively weaker connectivity with all clusters compared to other hippocampal ROIs (Supp. Fig. 6).

**Fig. 7.**
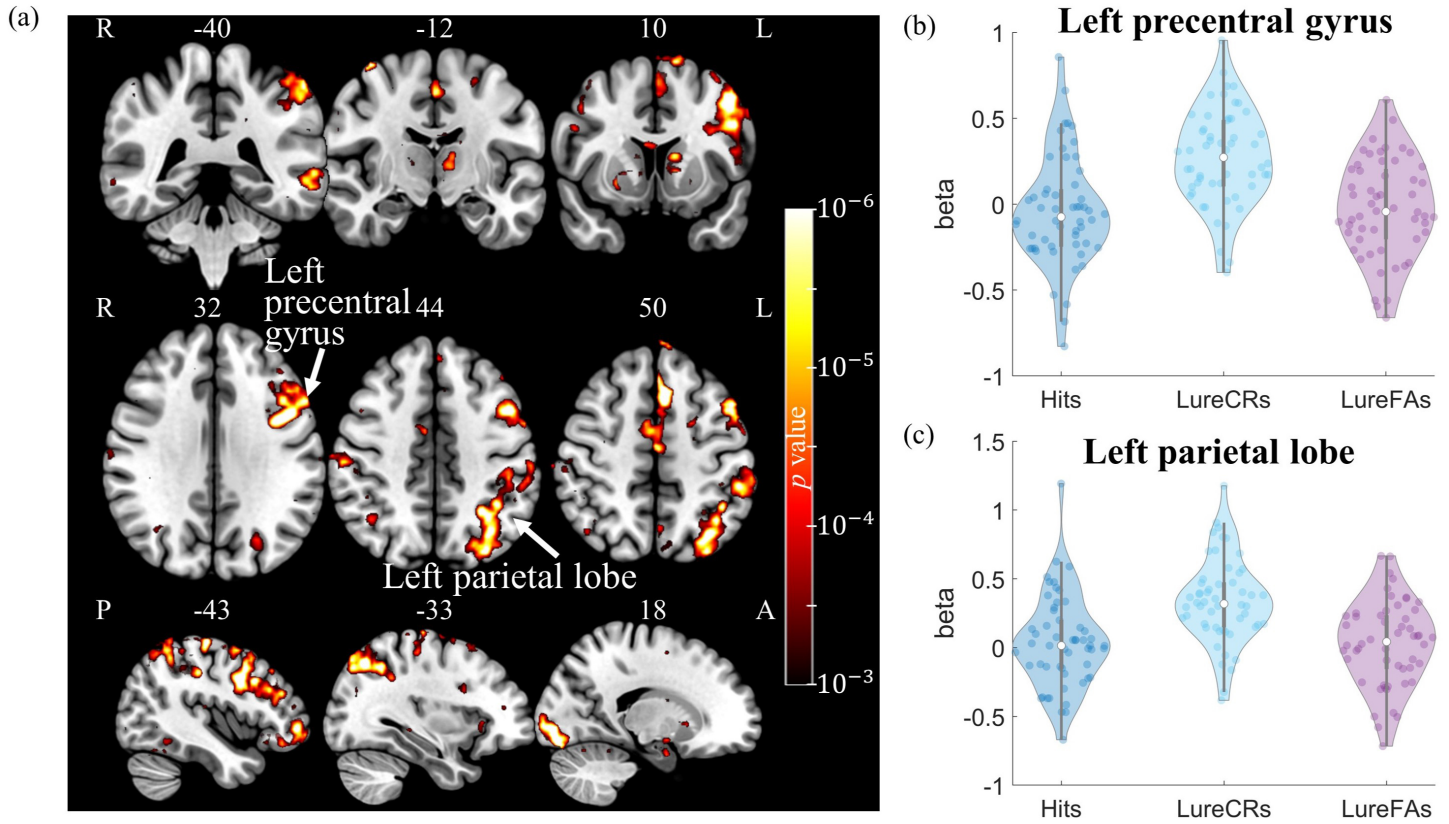
Comparisons of activity difference between LureCR and LureFA condition in the whole-brain analysis. (a) 3D view of the significance level identified from repeated-ANOVA analysis. Eleven clusters were found to be significant. Violin plots of beta values for the two largest clusters located at left precentral gyrus (b) and left parietal lobe (c) were shown in the figure. All the other clusters had similar activity patterns and are shown in theSupp. Figure 5.

## 4 Discussion

Determining the activity of hippocampal subfields and other brain areas from a single cohort is crucial for understanding the collective effect of these regions in the pattern separation process. By taking advantages of 7T MR scanner and advanced imaging protocols, our study collected whole-brain high-resolution fMRI data while participants were performing a visual object mnemonic similarity task. The pattern separation signal was observed to be left-lateralized and was present in various cortical and subcortical regions along with the hippocampus.

Consistent with the proposed role of CA3/DG in orthogonalizing overlapping representations in both young (Reagh & Yassa, 2014) and old population (Yassa, Lacy, et al., 2011), left anterior CA3/DG showed greater activity when lure objects were correctly identified as “similar” (e.g., LureCR) as opposed to “same” (e.g., LureFA) (seeFig. 5a), confirming that CA3/DG is involved in the pattern separation process. Interestingly, different from the observation among young adults (age range 18–29 years) (Stevenson et al., 2020), bilateral posterior CA3/DG did not show discriminative activity in our study. This lack of activity might be related to a diminished capacity for pattern separation and an increased propensity for pattern completion along aging (Wilson et al., 2006). In addition, BOLD signal overall might be weaker in older adults (Grady & Garrett, 2014), which made it more challenging to detect the subtle activity difference between LureCR and LureFA trials in hippocampus.

In the association analyses, the behavioral metric LDI was not significantly associated with age, which might be because our cohort was limited to elderly adults with age ranging from 61 to 81 years old. In addition, deteriorated lure discrimination with age (Yassa, Lacy, et al., 2011) might be more prominent for lure objects with smaller discrepancy (lure 1 and 2 in the MST task), which were not included in our task. Only including the lure objects with larger discrepancy (lure 3, 4, and 5 in the MST task, corresponding to LureS, LureM, and LureL in our study) could also explain higher lure correct rejection rate compared to a previous study (Yassa, Lacy, et al., 2011). The activity differences between LureCR and LureFA at left anterior CA3/DG and right anterior subiculum, but not at CA1, were positively associated with LDI, which further supports the involvement of CA3/DG in pattern separation (seeFig. 5b-c). The subiculum also plays a key role in mediating the interaction between hippocampus and cortical or subcortical regions (O’Mara et al., 2001), thus it is likely to be involved in various hippocampus-dependent processes, for example, pattern separation.

In the whole-brain analyses, across Hit, LureFA, and LureCR conditions, the frontoparietal network (seeFig. 6), particularly on the right hemisphere, was consistently demonstrated to have greater activity than the Foil condition, which was in line with previous findings, indicating the involvement of frontoparietal network in successful memory retrievals (Iidaka et al., 2006;Naghavi & Nyberg, 2005;Ramanan et al., 2019). Among participants with neurodegenerative disorders, delayed recall performance was correlated to the morphometry and structural connectivity of frontoparietal network (Ramanan et al., 2019), which supports the role of frontoparietal network in recalling previously viewed objects. Since in both Hit and LureFA conditions participants thought the same objects as in the encoding phase were presented, highly similar activity patterns for these two conditions were expected. Different from the right lateralized activity observed in LureFA and Hit conditions, LureCR condition showed activity in bilateral frontoparietal regions. Similarly, a previous study showed that bilateral parietal regions had greater activity in LureCR condition than Foil condition (Bowman & Dennis, 2016).

A comparison of whole-brain activity between LureCR and LureFA condition showed that LureCR had greater activity than LureFA in various cortical and subcortical areas. The significant clusters were mainly located in the left hemisphere (85.4% of voxels in left hemisphere), particularly the left frontoparietal network (62.3% of voxels). Such an activity difference was not observed in the right frontoparietal network. Evidence from visual search and selective attention tasks showed that frontoparietal regions could encode simple feature properties and then bias downstream sensory regions to represent those features based on attentional demands (Bichot et al., 2019;Ester et al., 2016). The additional recruitment of left frontoparietal network in the LureCR over LureFA condition indicates its neural activity engaged in identifying more unique features that distinguish between different objects. Similarly, a previous study showed that left-lateralized prefrontal regions preferentially supported lure rejection than correct target identification (“same” | target) (Bowman & Dennis, 2016). In addition, increased connectivity during lure rejection but not during target identification between the frontoparietal network and the hippocampus suggests the collective efforts of these two brain areas in pattern separation. The representation of unique features in the frontoparietal network could propagate to and be amplified at hippocampus, which was part of the proposed “cortico-hippocampal pattern separation” framework (Amer & Davachi, 2023). A substantial literature illustrated that the frontoparietal network contributed to functions similar to pattern separation, such as modulating the demands between competing memories or stimuli. The left ventrolateral prefrontal cortex has previously been shown to play a role in functions akin to pattern separation, such as resolving interference between competing memories (Amer et al., 2016;Badre & Wagner, 2007). In the MST, frontoparietal regions are likely activated to selectively focus attention and inhibit distraction (i.e., cognitive control) to make the right judgment in the given task.

Different from other brain networks (e.g., default mode network, sensorimotor network, auditory network and etc.) showing more symmetric spatial maps in brain network analysis, the frontoparietal network was frequently detected to be lateralized with separate right and left components (Rosazza & Minati, 2011), indicating that both temporal signatures and functions of left and right frontoparietal networks might not be fully symmetric. In addition, the frontoparietal network was observed to be activated in a 3-Tesla mnemonic discrimination task fMRI study, although the activity difference between LureCR and LureFA did not reach the significance level (Nash et al., 2021). The differences between our and their findings might be related to the improved MRI data quality at 7T, higher temporal resolution, and optimized task design. Furthermore, multiple studies have demonstrated the functional lateralization of regions in the frontoparietal network in various conditions, such as memory task (Golby et al., 2001), phasic alertness (Haupt et al., 2020), and drug treatment (Ye et al., 2021), which further supported the distinct roles of left and right frontoparietal networks in our study. Collectively, our result suggests that the right frontoparietal network primarily contributes to memory retrieval and the left frontoparietal network is mainly engaged in pattern separation. Together with the evidence of significant connectivity between hippocampal ROIs and clusters located at frontoparietal areas, these findings suggested that the hippocampus, or more broadly the temporal lobe, interacts with left frontoparietal regions for discriminating the lure objects in the recognition phase. By visually inspecting fMRI volume, severe distortions and signal dropouts were observed at part of the frontal lobe, possibly due to B0 inhomogeneity. However, the distorted area is not located at, or close to, the activation detected in the study (Supp. Fig. 7), indicating that the activation identified in the frontal lobe is unlikely due to the distortion artifact.

In the subcortex, clusters located at bilateral caudate nucleus and left thalamus showed activity of the pattern separation signature. Robust functional connectivity between subcortical regions and almost all hippocampal ROIs, particularly the anterior ones, was observed in our task. Previous evidence suggests that coordinated striatal-medial temporal activity may be essential for episodic memory processing (Murty et al., 2015;Sadeh et al., 2011). The subcortical regions potentially facilitated the hippocampal downstream signal passing to the frontal regions through the thalamus. Considering that the frontoparietal network also showed a pattern separation signature, it further supports that the hippocampus might interact or connect with corticostriatal loops (Pennartz et al., 2009) to discriminate lure objects. Alternatively, the functional demand from the hippocampus might shift toward the subcortical regions as part of the aging effect as observed in a virtual navigation task fMRI study (Konishi et al., 2013). If the functional shift is not limited to the subcortical regions, it might also explain the pattern separation signature observed in the cortical regions. Comparing the activity with a younger population performing the same task could be beneficial to clarify how age effect could impact the regions engaged in pattern separation. There were voxels located at bilateral hippocampus showing distinct activity between LureCR and LureFA condition at uncorrected p < 0.001 in the whole-brain analysis; however, these voxels did not pass the cluster-wise correction. The lack of significance might be because of the imperfect spatial transformation when normalizing to standard template space (i.e., MNI template in our study). Alternatively, the hippocampus itself is relatively small, and the clusters in this area were less likely to pass the cluster-wise correction.

There are a few limitations in the current study. First, the current study did not collect task fMRI data from a younger population. Therefore, it is uncertain if the regions identified in the study could be replicated with a group of younger participants. The activity observed in the cortex and subcortex might be due to function demand shifting from the hippocampus or functional reorganization with aging (Sala-Llonch et al., 2015). Second, the connectivity characterized by Pearson’s correlation did not reveal a clear pattern of the preferential connectivity between hippocampal ROIs and the significant clusters identified from whole-brain analysis. Future studies are warranted to refine the connectivity analysis, such as graph theory analysis and granger causality analysis, and determine the association of brain connectivity with behavioral metrics. Third, with the resolution of our fMRI data, it remains challenging to delineate CA3 from DG. Because of this reason, like previous MST fMRI studies, CA3 and DG are combined for the analysis. MST fMRI data with finer spatial resolution are needed to separately investigate the functional roles of CA3 and DG.

In summary, by using ultra-high field MR scanner and advanced fMRI protocol, our study revealed that the left frontoparietal network, in addition to CA3/DG, could be involved in discriminating similar visual objects, and the right frontoparietal network contributes to memory retrieval. The subcortical regions also have a “pattern separation” signature in our elderly cohort, but their role in the process requires more evidence to be clarified.

## Supplementary Material

Supplementary Material

## Data Availability

The data used in the study can be requested by sending a research proposal to the principal investigator (PI) Dr. Dietmar Cordes (email:cordesd@ccf.org) by using your institutional email address. The research proposal is 1-page maximum. Please provide a clear and informative title for your proposed research. Please briefly describe the overall rationale for your study and summarize the specific aims/hypotheses that you will test with the specific data elements you are requesting. The code used in this paper for image analysis has been developed and released viawww.github.com/CCLRCBH-BIC.
