# Supplementary material for: Pattern separation involves regions beyond the hippocampus in non-demented elderly individuals: A 7T object lure task fMRI study
Source: Imaging Neurosci (Camb). 2024 Dec 19;2:imag-2-00404. doi: 10.1162/imag_a_00404 (PMC11997958; doi:10.1162/imag_a_00404)
Supplement: Supplementary Material [file imag_a_00404-supp.pdf]

SUPPLEMENTAL MATERIAL

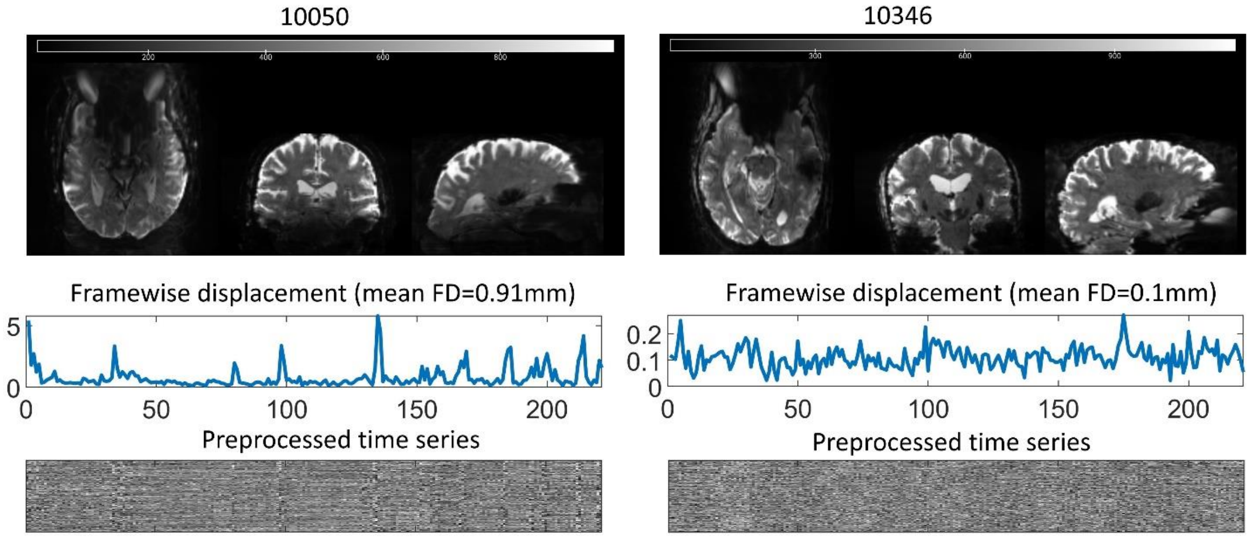

Supp. Figure 1: Example plots of task fMRI data during the recognition phase from two subjects. One subject has minimal motion with mean framewise displacement of 0.1 mm and the other subject has mean framewise displacement of 0.91 mm. Top panel: mean fMRI volume of the session. Middle panel: framewise displacement over time points. Bottom panel: Gray plot of preprocessed time series after slice-time correction, distortion correction, and realignment. The y-axis indicates voxels and x-axis indicates time point.

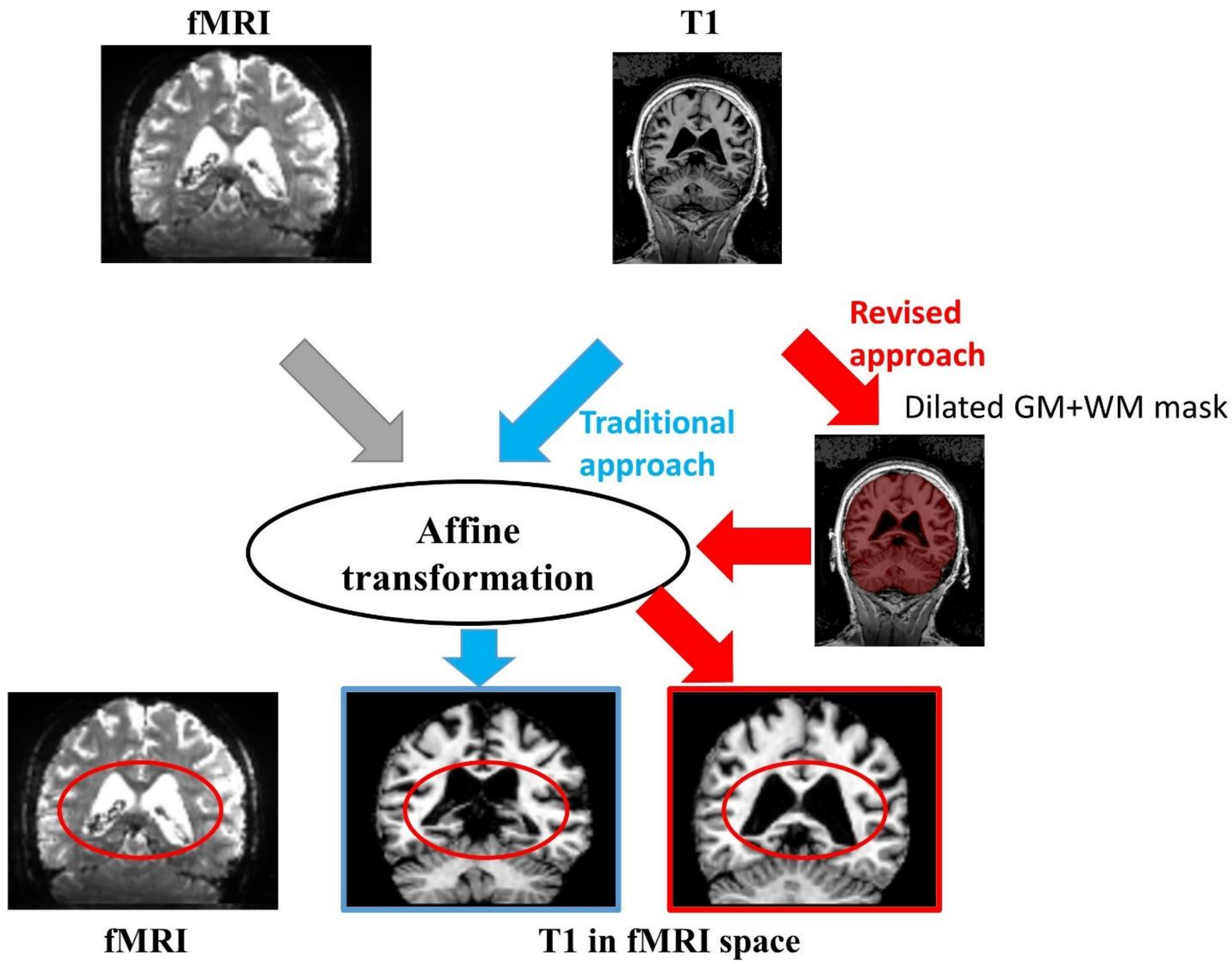

Supp. Figure 2: A comparison of revised and traditional approach for affine registration between fMRI and T1 images. Different from traditional approach (blue arrows), a dilated GM+WM mask is fed to the affine transformation in the revised approach (red arrows), which consistently showed better performance with our 7T fMRI data.

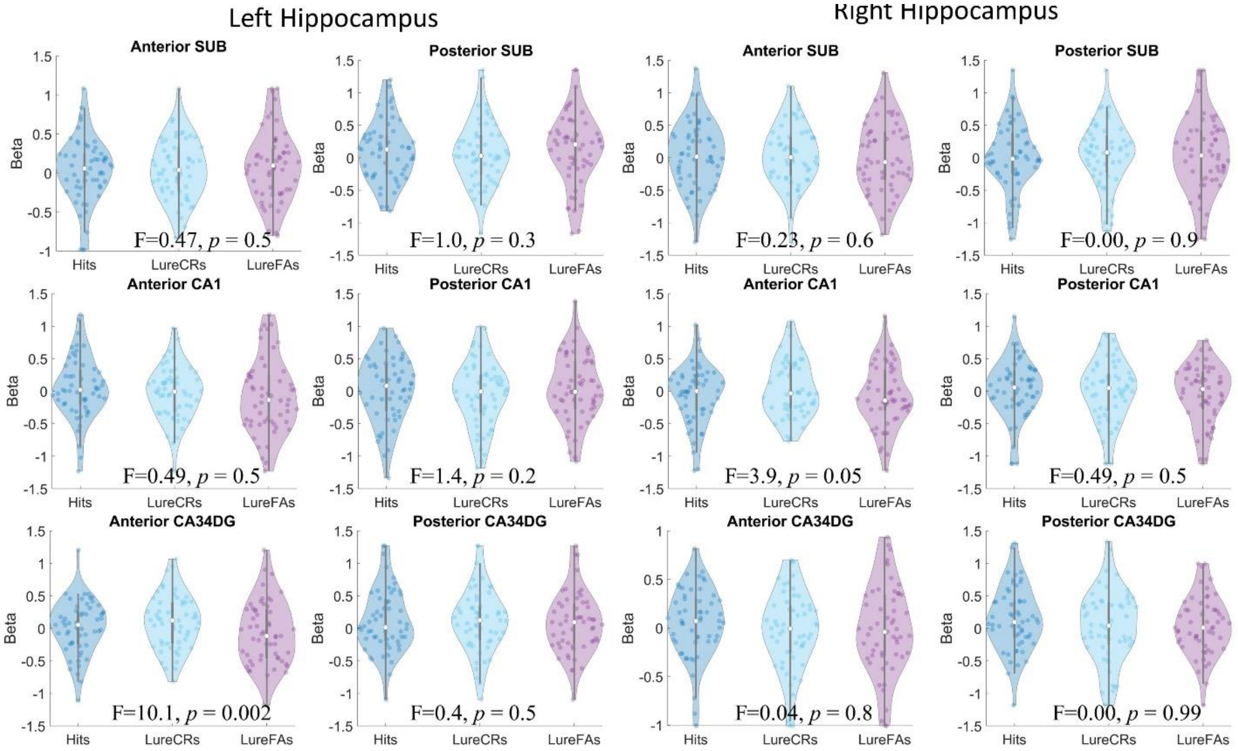

Supp. Figure 3: Beta coefficients of all hippocampal ROIs for Hit, LureCR, and LureFA conditions. The significance levels from repeated-ANOVA (uncorrected p value) were marked in the figure.

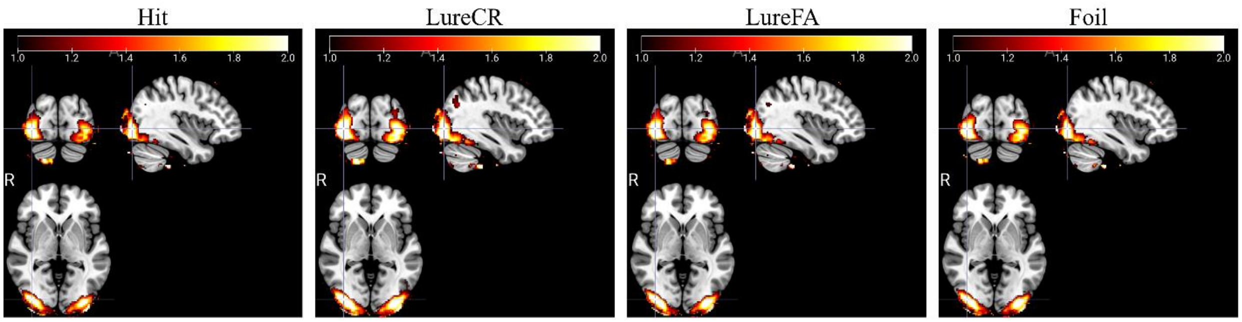

Supp. Figure 4: Mean beta values of Hit, LureCR, LureFA, and Foil conditions from whole brain analysis. The coefficients before treating Foil condition as baseline condition are shown in the figure.

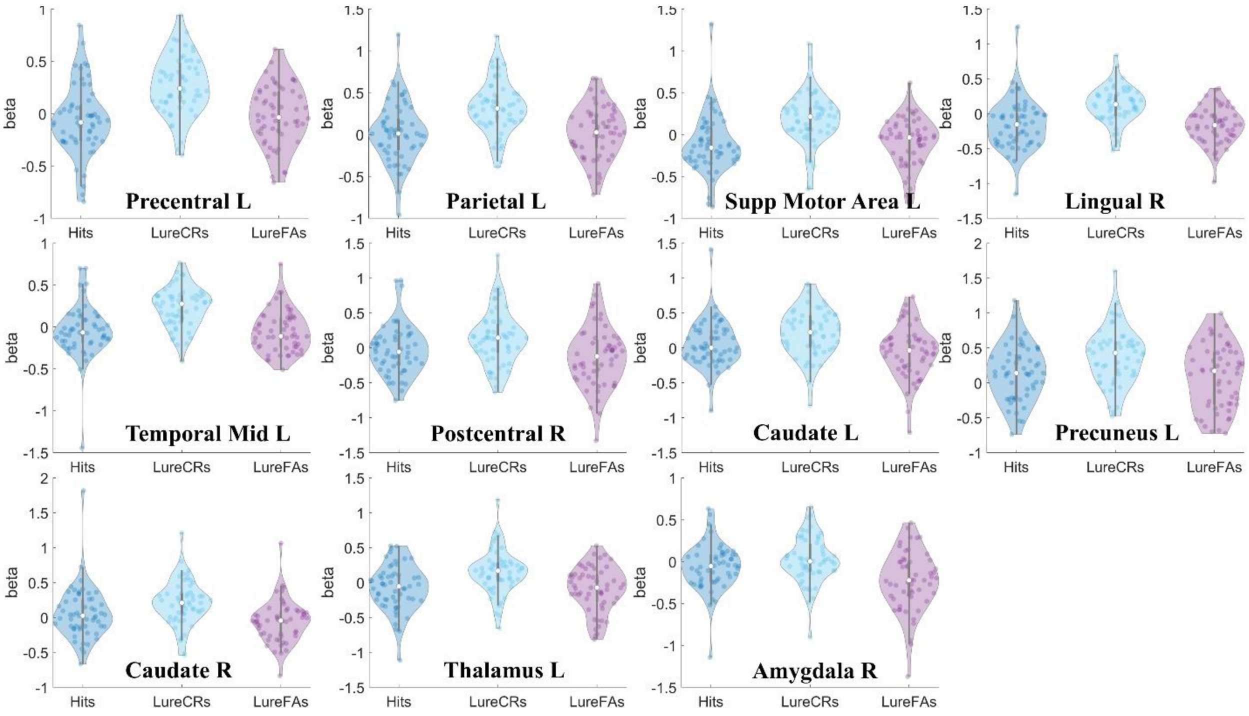

Supp. Figure 5: Beta coefficient for the 11 significant clusters identified from the whole brain analysis. The beta coefficient for each cluster is computed by averaging the beta coefficients of all voxels in the cluster. The clusters from 1 to 11 in ascending order are located at left precentral gyrus (2983 voxels), left parietal lobe (2478 voxels), left supplementary motor area (861 voxels), right lingual gyrus (854 voxels), left middle temporal lobe (735 voxels), right postcentral gyrus (204 voxels), left caudate nucleus (168 voxels), left precuneus (151 voxels), right caudate nucleus (135 voxels), left thalamus (114 voxels), and right amygdala (84 voxels).

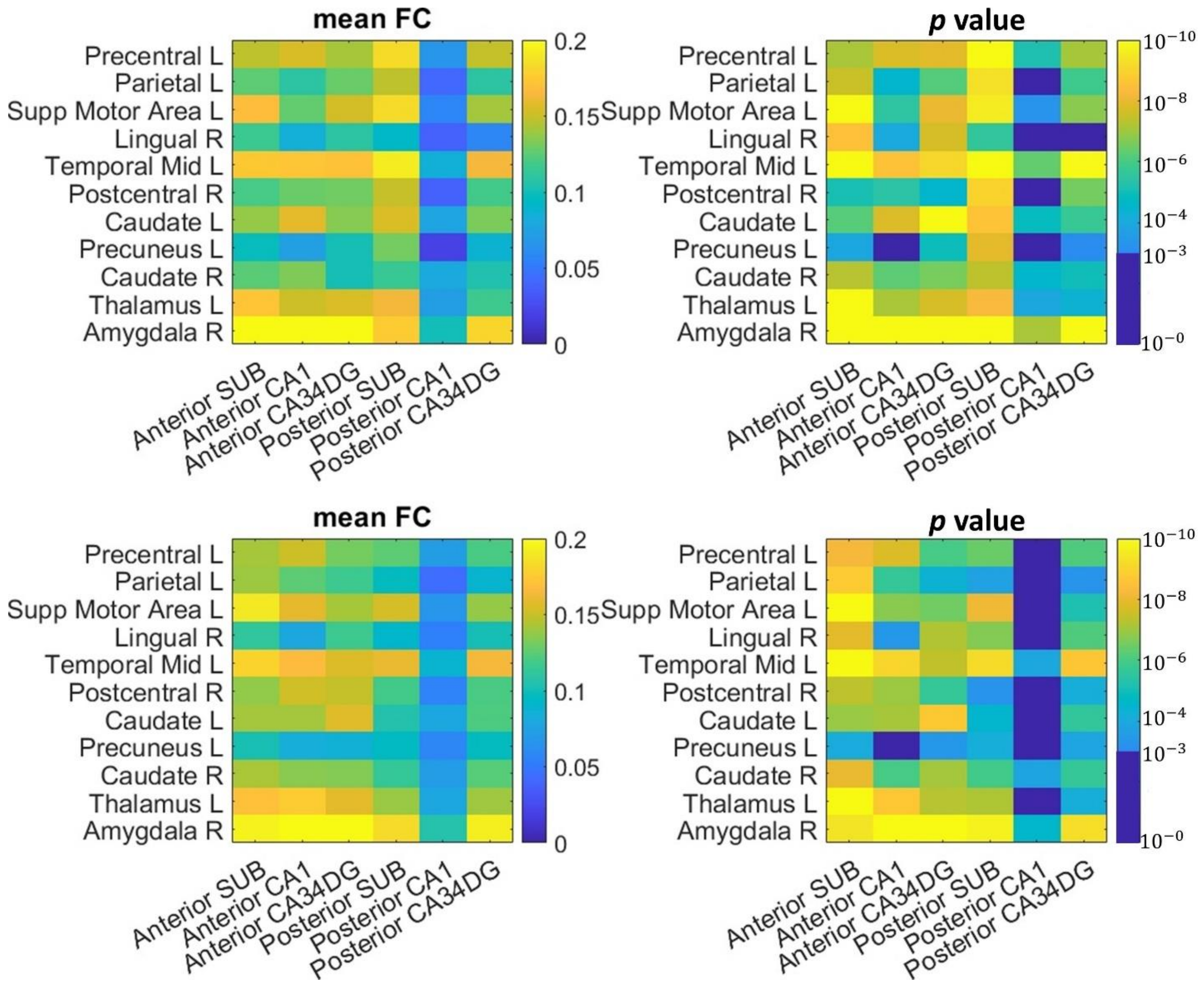

Supp. Figure 6: Functional connectivity between the significant clusters identified from whole brain analysis and the hippocampal ROIs. Functional connectivity is characterized by Pearson's correlation. (a) Mean functional connectivity across all participants. (b) Significance level of the functional connectivity based on one-sample t-test after Fisher r-to-z transformation. The connectivity that did not reach the significance level of p=0.001 was marked as dark blue in the figure.

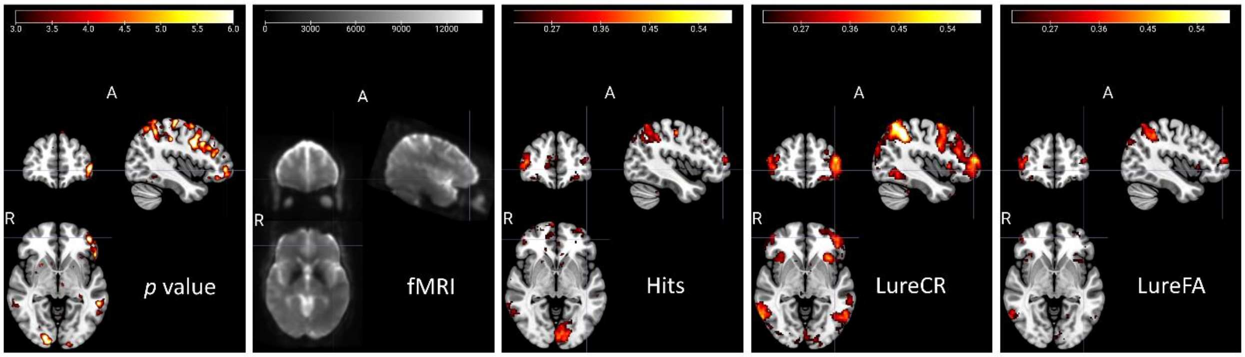

Supp. Figure 7: The 3D representation of p value map from ANOVA, fMRI image, beta coefficient for Hits, LureCR, and LureFA (from left to right). Similar images can be found for other subjects.
